# In silico identification of novel chemical compounds with antituberculosis activity for the inhibition of InhA and EthR proteins from Mycobacterium tuberculosis

**DOI:** 10.1016/j.jctube.2021.100246

**Published:** 2021-05-26

**Authors:** Sajal Kumar Halder, Fatiha Elma

**Affiliations:** a Department of Biochemistry and Molecular Biology, Jahangirnagar University, Savar, Dhaka 1342, Bangladesh; b Research assistant at Padma Bioresearch, Dhaka, Bangladesh

**Keywords:** Tuberculosis (TB), Drug-likeness, ADMET analysis, Molecular docking and Molecular dynamics simulations, Drug discovery

## Abstract

Tuberculosis (TB) continuously poses a major public health concern around the globe, with a mounting death toll of approximately 1.4 million in 2019. Reduced bioavailability, elevated toxicity, increased side effects, and resistance of multiple first-line and second-line TB medications, including isoniazid, ethionamide necessitate studies of new drugs. The method of computational biology and bioinformatics approach allows virtual screening of a large number of drugs, reduces growing side effects of medications, and predicts potential drug resistance over time. In this study, we have analyzed fifty small molecules with antituberculosis properties using in silico approach including molecular docking, drug-likeness assessment, ADMET (absorption, distribution, metabolism, excretion, toxicity) profile evaluation, P450 site of metabolism prediction, and molecular dynamics simulation. Among those fifty compounds, 3-[3-(4-Fluorophenyl)-1,2,4-oxadiazol-5-yl]-N-(2-methylphenyl) piperidine-1-carboxamide (C22) and 5-(4-Ethyl-phenyl)-2-(1H-tetrazol-5-ylmethyl)-2H-tetrazole (C29) were found to pass the two-step molecular docking, P450 site of metabolism prediction and pharmacokinetics analysis successfully. Their binding stability for target proteins has been evaluated through root mean square deviation and root mean square fluctuation, Radius of gyration analysis from 10 ns Molecular Dynamics Simulation (MDS). Our identified drugs (C22 and C29) performed better than the control drugs (Isoniazid, Ethionamide) regarding binding affinity and molecular stability with the regulatory proteins (InhA, EthR) of Mycobacterium tuberculosis. The study proposed these compounds as effective therapeutic agents for Tuberculosis drug discovery, but further in vitro and in vivo testing are needed to substantiate their potential as novel drugs and modes of action.

## 1 Introduction

Tuberculosis is among the earliest known infectious diseases [1]. Since the introduction of Mycobacterium tuberculosis as the causative agent of tuberculosis by Robert Koch in 1882, it has grown and evolved as one of the fatal pathogens in the history of infectious disease in humans [2]. Mycobacterium tuberculosis is a distinct acid-fast, slowly developing, aerobic bacteria with a size of 0.8–4 µm that belongs to the Mycobacteriaceae family [3]. The most regularly studied strain H37Rv of M. tuberculosis has a genome composed of 4.4 Mb that encodes 4018 genes with an average G + C content of 65.6% [4].

Tuberculosis (TB) is a communicable disease, transmitted by a person suffering from active TB with the release of small nuclei respiratory droplets (ranges between 0.65 to greater than 7.0 μm) containing viable airborne bacteria [5], [6]. Once the bacilli reach the lung alveoli, either the mycobacteria are phagocytized by mature alveolar macrophages, or the active mycobacteria start reproducing within the macrophage, which then causes their lysis [7]. This local inflammation attracts monocytes from neighboring blood vessels and leads to granuloma formation by encapsulating bacilli for controlled progression (LTBI) [8]. In case of failure, the infection starts to spread through both lymphatic channels and blood circulation which can cause serious damage to the lungs (pulmonary TB), along with other regions of the body, i.e., brain, spinal cord, lymph node, abdomen, bones/joints, intestinal and genitourinary system [7], [9], [10], [11]. Active TB symptoms depend on the severity of the spread; generally, some of the clinical features are persistent coughing for more than 3 weeks, bloody sputum (hemoptysis), chest pain, fever, fatigu e/weakness, weight loss, anorexia, breathlessness, etc. The person with latent tuberculosis infection (LTBI) presents no symptoms and cannot transmit Mtb to others, but may develop active TB [12].

One of the major barriers to therapeutic interference against *Mtb* is its unusual cell envelope structure that has a high lipid content with unique long-chain highly hydrophobic α‐alkyl‐β‐hydroxy fatty acids, mycolic acids (MAs) [13], [14]. MA functions to maintain the structural integrity and viability of the mycobacterium by acting as an extraordinarily efficient permeability barrier and contributes to the intrinsic resistance towards host bactericidal agents or different classes of antibiotics [15], [16], [17], [18]. It also protects against oxidative stress, elicits differentiation of macrophages into foamy macrophages [19], [20]. Therefore, in our in-silico approach, we chose to target a pivotal enzyme, enoyl-acyl carrier protein reductase (InhA) involved in the synthesis of MA [21] and EthR, a negative transcriptional regulator which reduces the expression of EthA enzyme [22]. The inhA gene encodes NADH-dependent enoyl-acyl carrier protein (ACP)-reductase enzyme which is associated with long chain fatty acids (mycolic acids) synthesis and reduction phase of fatty acid production. Mutation of the inhA gene facilitates resistance to several first-line drugs such as isoniazid, making it an appropriate target for drug discovery [23]. EthR belongs to the tetR/CamR family of transcriptional repressor that negatively regulates the synthsis of EthA enzyme which is involved in the activation of thiocarbamide-bearing drugs ethionamide. Low activation of ethionamide by the EthA enzyme increases the risk of Mycobacterium tuberculosis becoming resistant to this type of drugs [22], [24]. This mechanism implies its significance as therapeutic target for resistance to these drugs.

WHO recommended a standard 6-month administrative treatment for active, pulmonary, and pharmaco-sensitive TB with different combinations of four first-line medications: isoniazid, rifampin, ethambutol, and pyrazinamide as bases for treatment [10], [25]. Unfortunately, the complex long-term administrative duration leads to non-compliance of treatment leading to failure and relapse which causes the bacterium to become resistant over time to anti-TB medications [26]. The antibiotic-resistant strains of Mycobacterium tuberculosis are sabotaging conventional tuberculosis therapy due to resistance to a wide range of anti-TB medications. Primary and secondary resistance contributes to the transfer of resistant strains to newer hosts and the development of drug resistance to two or more drugs respectively [27]. Resistance in this bacterium includes overexpressed drug targets due to mutation of repressor or promoter region of targets, alteration of drug targets by enzymatic process, cancelation of mechanism of prodrugs such as Ethionamide that induces inactivation of first-line drugs like Isoniazid, direct inactivation of drugs within the bacterial cell by enzymatic process, drug efflux process through which drugs are transferred outside the bacterial cell [28]. The onset of MDR-TB or multidrug resistance TB exacerbated the treatment of TB because of its poor efficacy, toxicity also the extra cost of about 20 months of extended administration of second-line drugs [29]. Furthermore, in 2016 an estimated 6.2% incidence of extensively drug-resistant TB (XDR-TB) among people with MDR-TB cases around the world has made the treatment more complex with limited efficacy of existing drugs [30], [31].

Although TB is a treatable infection, the massive emergence of resistance to antibiotics makes it a global threat. It is undeniable that the world needs shorter and simpler yet affordable, effective, tolerable, and safe new drug regimens to improve the current treatment of TB to get around the escalation of drug resistance. Our in-silico approach seeks to offset the burden of existing drug resistance scenarios through the exploration of new drug performance. As a part of the experiment, a total of fifty drug-like compounds were collected to assess their effectiveness in the treatment of TB and clinical management. Our experiment proceeded through evaluating the binding affinity of fifty ligands utilizing two different docking tools DockThor server and Autodock vina software individually against the InhA (PDB ID: 3FNG) and EthR (PDB ID: 3G1M) receptor proteins. Identification of metabolic sites and pharmacokinetics and pharmacodynamics properties of tested compounds contributed to confirm their drug-like properties. Molecular dynamics simulations were conducted to determine the molecular stability of the best drug-like compounds with respective receptor complexes.

## 2 Materials and methods

### 2.1 Molecular docking

#### 2.1.1 Initial molecular docking in DockThor program

The crystal structures of two receptors i.e., Crystal structure of InhA bound to triclosan derivative (PDB ID: 3FNG) and EthR from Mycobacterium tuberculosis in complex with compound BDM31381 (PDB ID: 3G1M), were taken from Protein Data Bank (https://www.rcsb.org/search). Fifty compounds were collected with anti-TB properties from both ACD: Antibacterial Chemotherapeutics Database (http://amdr.amu.ac.in/acd/index.jsp) and PubChem (https://pubchem.ncbi.nlm.nih.gov/) servers. Isoniazid (INH) and Ethionamide (ETH) were used as control drugs against InhA and EthR proteins respectively. In the beginning, the PDB structures were modified using PyMOL tools (PyMOL) by clearing the water molecules from the structure [32] and then minimizing the structure employing Swiss-PdbViewer [33]. The preliminary docking of two receptor protein and fifty ligands were carried out by DockThor docking server (https://www.dockthor.lncc.br/v2/). The algorithm of the program is based on flexible-ligand and rigid-receptor grid-based system [34].

#### 2.1.2 Autodock-vina binding affinity prediction

Following the initial affinity prediction, desired ligands with lower affinity scores than control drugs were chosen to evaluate the bound conformation and binding energy. Autodock vina (AutoDock 4.2) was used to evaluate bound conformation and binding energy of those selected ligands. This tool uses the Lamarckian Genetic Algorithm to evaluate the binding energy of ligands with receptor proteins. After protein and ligand preparation, they were converted to vina-compliant PDBQT format using AutoDockTools-1.5.6rc3 [35]. The same tool was used to prepare grid boxes with preferred dimensions. The center of the grid box for InhA protein (PDB ID: 3FNG) was fixed where X = 22.466, Y = 50.786, Z = -11.275 with a dimension of 92 × 72 × 68 Å and for EthR protein (PDB ID: 3G1M) the coordinate was fixed X = 33.206, Y = 69.92, Z = 10.822, with a fixed dimension of 58 × 84 × 50 Å. CASTp server [36] was exploited to determine ligand binding sites of proteins and calculate grid box size.

### 2.2 P450 site of metabolism (SOM) prediction

The most promising four compounds were chosen for further calculation. The probable sites of metabolism of these compounds were predicted using RS-Web Predictor 1.0 (http://reccr.chem.rpi.edu/Software/RSWebPredictor/) [37]. This prediction tool uses 3A4, 1A2, 2A6, 2B6, 2C8, 2C9, 2D6, 2E1, 3A4 CYP isoforms for cytochrome P450 site evaluation.

### 2.3 Drug-likeness properties analysis and ADMET prediction

Determination of drug-likeness property of drug-like compounds is one of the vital steps of drug discovery. Prediction of these properties was completed utilizing Lipinski’s rule of five [38], Ghose’s rule [39], Veber’s rule [40], Muegge’s rule [41], TPSA, and No of rotatable bonds. The calculation was carried out using SwissADME online tool (http://www.swissadme.ch/index.php) [42].

ADMET properties of each of four compounds were predicted using another online tool, admetSAR (http://lmmd.ecust.edu.cn/admetsar2/) [43], [44]. In each of these prediction studies, canonical smiles of the compounds were used from the PubChem database (https://pubchem.ncbi.nlm.nih.gov/).

### 2.4 Visualization and interaction analysis

Visualization of the non-bonded relationship of 2D and 3D conformation of protein–ligand docked complexes was done by BIOVIA Discovery Studio 4.1 Visualizer [45]. This tool was utilized to get the total number of hydrogen bonds and interacting amino acids of proteins with respective ligands.

### 2.5 Molecular dynamics simulation

**Fig. 1:**
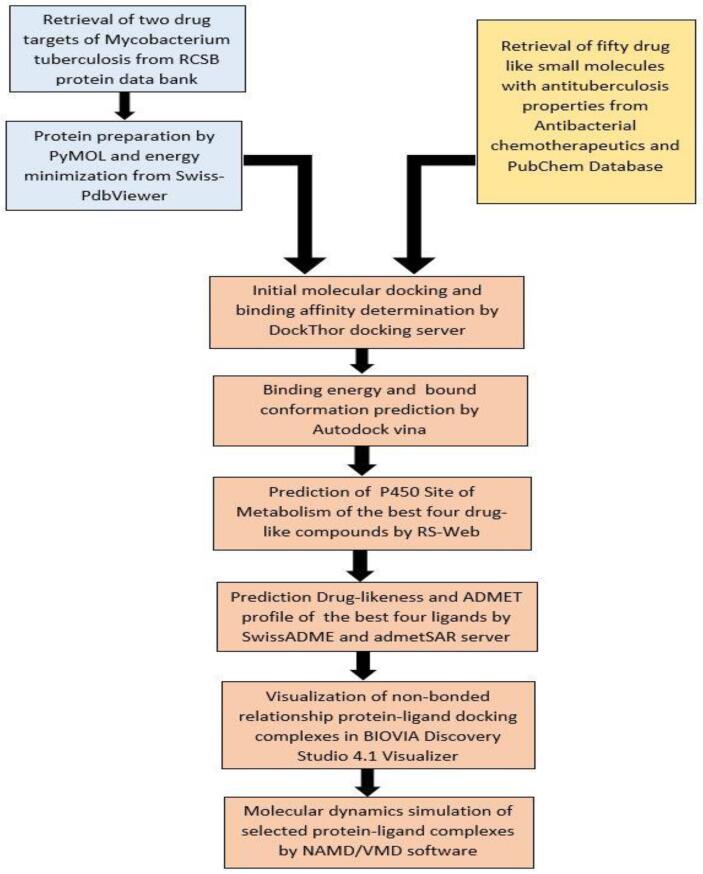
Complete methodology of this study in a concise flowchart.

The study of molecular dynamics simulation (MDS) is a thermodynamics-based operation that helps to study the dynamic perturbation found in the protein–ligand complexes. In our experiment, to ensure the stability of protein-ligand complex, we subjected the best ligands screened from previous steps to the molecular dynamics simulation (MDS) study with their respective proteins. We simulated the docking complexes using the NAMD_2.14bNAMD_2.14b2_Win64-multicore-CUDA version [46] implying CHARMM 36 force field [47] and TIP3P water model. A multi-step time algorithm was used, with an integration time step of 2 femto seconds. Visual molecular dynamics (VMD) [48] was used to generate psf files of protein–ligand complexes, water box and for neutralizing the system with sodium (Na+) and chloride (Cl-) ions. Ligand topology and parameter files were generated using the CHARMM-GUI web service [49]. The simulation was run for 10 ns, where the system was minimized for 1000 steps. Langevin thermostat was used to maintain a constant temperature of 310 k. Periodic boundary conditions were applied surrounding the system. The complete workflow of our methodology was summarized in Fig. 1.

## 3 Results

### 3.1 Molecular docking analysis

To filter out the best ligands from molecular docking analysis, two separate docking tools were used with a different algorithm. Initial docking results of the DockThor server were further scanned by Autodock-vina software. Detailed information of all the collected drug-like compounds was provided in Supplementary material.

Total fifty ligands were primarily docked individually against the InhA and EthR receptors via the DockThor server. Against target proteins, InhA and EthR, all the compounds showed better affinity than control 1 (Isoniazid) and control 2 (Ethionamide). The binding affinity score between InhA, EthR receptors, and fifty ligands, as well as control drugs, is depicted in Table 1.

**Table 1:** Binding affinity score between receptors (InhA, EthR) and fifty ligands, along with control drugs.

Drug Identifier	Drug Name	Binding Affinity (kcal/mol)	
		InhA (3fng)	EthR (3g1m)
Control 1	Isoniazid (INH)	−5.50	–
Control 2	Ethionamide (ETH)	–	−6.20
C1	1,2-Benzisothiazol-3(2H)-one	−7.55	−7.52
C2	Tetrathiafulvalene	−8.10	−6.68
C3	5-Chloroindoline	−7.79	−8.49
C4	N-Tert-butyl-2-phenylacetamide	−7.82	−7.35
C5	5-Oxo-2,3,5,9b-tetrahydro-thiazolo[2,3-a]isoindole-3-carboxylic acid	−6.48	−6.54
C6	Bis(4-hydroxyphenyl)disulfide	−7.57	−7.18
C7	5-(4-Methoxyphenyl)-2H-tetrazole	−7.83	−8.51
C8	7-Bromo-6-hydroxy-2,3-dihydro[1]benzothieno[2,3-d]pyrrolo[1,2-a]pyrimidin-10(1H)-one	−9.19	−7.43
C9	4-Benzoylphthalic acid	−8.62	−6.80
C10	1,1′-Ethanediyl-bis-cyclopentanol	−8.06	−7.00
C11	3-(3-Chlorophenyl)-1,1-diethylurea	−8.78	−7.67
C12	(3,4-Dimethoxy-benzyl)-thiazol-2-yl-amine	−8.52	−7.13
C13	3-(Diisopropyl-phosphinoyl)-benzoic acid	−6.38	−6.33
C14	N-[2-(4-Fluoro-benzoyl)-benzofuran-3-yl]-acetamide	−9.22	−8.03
C15	1-Adamantyl-(4-hydroxy-4-pyridin-3-ylpiperidin-1-yl)methanone	−8.64	−8.13
C16	N-(Furan-2-ylmethyl)-4-phenacylthieno[3,2-b]pyrrole-5-carboxamide	−9.09	−8.25
C17	Carbenicillin	−6.90	−6.31
C18	N-[1-(Furan-2-ylmethylamino)-3-methyl-1-oxobutan-2-yl]-2-[(4-methoxybenzoyl)amino]benzamide	−8.72	−7.54
C19	4,6-Bis(propan-2-ylamino)-1,3,5-triazine-2-carboxamide	−6.58	−6.25
C20	3-Phenyl-N-(2,2,6,6-tetramethylpiperidin-4-yl)propanamide	−7.28	−7.09
C21	3-Methyl-benzofuran-2-carboxylic acid pyridin-4-ylamide	−9.16	−8.17
C22	3-[3-(4-Fluorophenyl)-1,2,4-oxadiazol-5-yl]-N-(2-methylphenyl)piperidine-1-carboxamide	−9.62	−8.51
C23	2-(Piperazin-1-yl)-4,6-di(pyrrolidin-1-yl)-1,3,5-triazine	−8.14	−6.87
C24	2-[2-(4-Methoxy-phenyl)-thiophen-3-yl]-propionic acid	−8.84	−6.88
C25	1-Phenylmethanesulfonyl-piperidine-3-carboxylic acid (2,3-dihydro-benzo[1,4]dioxin-6-yl)-amide	−7.10	−7.59
C26	4-Cyclohexylaminomethyl-1H-quinolin-2-one	−8.15	−7.50
C27	(Naphthalen-1-ylcarbamoylmethylsulfanyl)-acetic acid	−8.22	−6.87
C28	2-Propylamino-nicotinamide	−7.30	−6.73
C29	5-(4-Ethyl-phenyl)-2-(1H-tetrazol-5-ylmethyl)-2H-tetrazole	−8.56	−6.88
C30	Methyl 2-[4-(methylamino)-6-morpholin-4-yl-1,3,5-triazin-2-ylthio]acetate	−7.85	−7.02
C31	Oxostephanine	−9.42	−7.73
C32	Ergosterol peroxide	−7.95	−7.58
C33	Sanguinarine	−9.46	−8.97
C34	Micromeline	−9.33	−6.87
C35	Oleanolic acid	−6.79	−7.21
C36	Ursolic acid	−7.48	−7.83
C37	Plumbagin	−7.90	−7.56
C38	Maritinone	−8.43	−7.27
C39	Rutin	−7.71	−7.18
C40	Aloe emodin	−8.29	−7.08
C41	Epigallocatechin	−9.06	−6.63
C42	Umckalin	−8.61	−7.13
C43	Butein	−8.40	−6.69
C44	Luteolin	−9.15	−6.69
C45	2-Hydroxy-4-methoxybenzaldehyde	−7.63	−6.41
C46	Isoliquiritigenin	−8.34	−6.78
C47	Piperine	−9.18	−8.41
C48	Tiliacorinine	−8.33	−7.66
C49	Isobavachalcone	−9.36	−7.04
C50	Turgorin	−6.49	−6.86

After initial docking from the DockThor server, all the ligands were considered for computing binding energy and bound conformation with the assistance of Autodock Vina software. Two ligands were preferred over all ligands considering their lowest binding score towards InhA protein. Likewise, two ligands with the lowest binding score towards EthR protein were chosen for further analysis. Comparative Docking results of the Autodock-vina tool are listed in Table 2.

**Table 2:** Molecular docking results and Hydrogen bond interaction between receptors (InhA, EthR) and fifty ligands, along with control drugs by Autodock Vina.

	InhA (3FNG)			EthR (3G1M)		
Drug identifier	Binding energy (kcal/mol)	Number of hydrogen bonds	Interacting hydrogen bonds with receptor (H-bonds lowest distance (A˚)	Binding energy (kcal/mol)	Number of hydrogen bonds	Interacting hydrogen bonds with receptor (H-bonds lowest distance (A˚)
Control 1	−5.50	Four	ILE21 (1.98) SER94 SER94 SER94	–	–	–
Control 2	–	–	–	−6.30	Two	ASN179 (2.22) TRP145
C1	−6.20	None	None	−5.20	None	None
C2	−6.90	Two	TYR158(1.98) LYS165	−7.20	Two	ASN179 (2.58) ASN176
C3	−6.40	One	LEU63(2.55)	−6.70	One	ASN176 (2.79)
C4	−7.30	One	GLY96 (2.40)	−5.20	None	None
C5	−8.10	Two	GLY96 (2.21) GLY14	−5.90	Two	TYR148 (2.12) ASN93
C6	−7.00	One	ASP64(2.40)	−5.40	None	None
C7	−7.50	None	None	−7.70	None	None
C8	−7.40	Two	THR162 MET155 (2.13)	−6.60	Two	GLU180 (2.20) GLN125
C9	−9.20	One	ILE194 (2.17)	−7.10	Five	GLN125 ARG128 ARG181 (2.08) ARG181 ARG181
C10	−6.60	One	ILE194 (2.54)	−8.60	One	ASN176 (2.04)
C11	−6.30	Four	GLY96 (2.19) GLY96 ILE95 GLY14	−6.60	Two	MET102 (2.92) MET102
C12	−6.60	One	ILE194 (3.44)	−5.30	One	ASN93 (2.20)
C13	−7.30	Four	VAL 65 (2.27) ASP 64 GLY14 PHE41	−5.70	One	PHE184 (2.75)
C14	−9.50	Two	PHE41 (2.45) GLY14	−7.40	Two	TYR148 (2.35) LEU90
C15	−8.40	One	ASP64 (3.52)	−7.80	Three	TYR148 (1.72) TRP103 TRP103
C16	−6.80	Four	ASN159(2.45) ASN159 ASN159 ASN106	−6.20	Four	TYR148 (2.35) ALA91 ASN93 TYR148
C17	−8.70	Three	LYS165(2.22) ILE194 ILE95	−7.00	Four	TYR148 ARG159 GLU156 LEU90 (2.44)
C18	−9.30	Eight	ILE21 ALA22 (2.16) SER94 GLY96 GLY96 GLY96 GLY14 SER94	−8.20	Three	TYR148 (1.96) PRO94 PRO94
C19	−6.20	Four	ILE21(2.08) GLY96 GLY14 SER94	−6.00	Four	TYR148 (2.11) ALA91 PRO94 PRO94
C20	−8.70	One	PHE149 (2.60)	−8.20	One	ASN93 (2.76)
C21	−8.70	One	GLY14 (3.55)	−10.10	Three	ASN179 (2.24) ASN176 ILE107
C22	−10.40	One	LYS164 (2.63)	−7.50	Three	ALA91 TRP103 GLY106 (3.06)
C23	−7.20	Four	GLY14 (3.41) SER94 SER94 GLY96	−6.50	One	MET102 (3.12)
C24	−6.40	Two	GLY96 (2.14) GLY96	−5.90	None	None
C25	−9.80	Three	TYR158 (1.92) LYS165 GLU219	−6.50	Three	TYR148 (1.77) ALA91 THR97
C26	−9.30	Three	GLY14 LEU63 (2.19) SER13	−6.70	Three	MET102 (2.20) PRO94 ALA91
C27	−8.40	Four	ALA22 (2.16) SER94 THR196 SER20	−5.90	Two	LEU42 (2.33) ALA43
C28	−6.00	None	None	−5.40	Three	TYR148 ASN93 (2.12) ALA91
C29	−8.90	Three	LYS165 ASP148 (2.24) PRO193	−9.70	Two	MET102 (2.67) TRP103
C30	−6.50	Three	ALA22 (2.24) SER94 ASP148	−5.70	One	MET102 (3.78)
C31	−7.90	None	None	−7.50	Four	GLN125 (2.22) GLN125 ARG128 GLU180
C32	−9.30	None	None	−7.10	None	None
C33	−9.20	Two	ASN159 MET155 (3.23)	−8.00	One	ARG122 (3.04)
C34	−8.60	None	None	−6.10	Seven	TYR148 (1.97) LEU90 ASN93 PRO94 ALA151 ALA91 ALA91
C35	−8.70	One	ALA154 (2.26)	−6.70	Two	THR97 (2.16) PRO94
C36	−8.60	None	None	−7.30	None	None
C37	−8.30	Two	VAL65 (2.03) LEU63	−7.90	None	None
C38	−10.40	Two	THR39 (2.78) ALA198	−6.10	Two	ASN93 GLU156 (2.24)
C39	−9.60	Four	LYS165 ASP64 GLY14 (1.83) SER94	−6.90	Five	TYR148 ARG159 GLU156 (2.08) MET102 PRO94
C40	−8.80	Two	GLY14 (2.48) ILE15	−6.90	Two	TYR148 (2.11) TYR148
C41	−8.30	One	ILE194 (2.83)	−7.20	Four	GLN125 (2.08) ARG128 ARG181 GLU190
C42	−6.70	Three	TYR158 (2.02) LYS165 PRO193	−6.80	Two	GLU190 (1.98) ARG122
C43	−8.70	Two	ALA191 PRO156 (2.49)	−7.80	Three	ASN179 (2.14) ASN176 GLY106
C44	−9.20	Two	ILE194 (2.76) GLU219	−4.80	Two	GLY106 LEU90 (3.49)
C45	−5.50	Three	ILE194 ILE194 (2.05) ILE194	−6.10	Six	TYR148 ALA91 LEU90 PRO94 (1.80) MET102 THR97
C46	−8.80	One	PRO156 (2.22)	−6.80	Four	GLN125 (2.12) ARG181 ARG181 ARG181
C47	−9.20	One	LYS165 (2.30)	−6.10	Three	ARG159 (2.51) LEU90 LEU87
C48	−9.30	One	GLY14 (3.69)	−8.20	Four	GLN125 GLU180 (2.03) ARG181 GLU180
C49	−8.60	One	ALA22 (2.32)	−9.30	One	GLY14 (3.69)
C50	−8.30	Five	GLY14 ILE21 ALA22 (1.92) SER94 SER94	−5.60	Six	TYR148 (2.07) TYR148 ARG159 GLU156 ALA91 PRO94

### 3.2 P450 site of metabolism (SOM) prediction

Cytochromes P450 is key to metabolizing foreign materials like drugs. Identification of metabolic sites in drug-like molecules determines their ability to be excreted from the body. The probable metabolic sites of CYP (1A2, 2A6, 2B6, 2C8, 2C9, 2C19, 2D6, 2E1, 3A4 and combined) of the four ligands: -3-[3-(4-Fluorophenyl)-1,2,4-oxadiazol-5-yl]-N-(2-methylphenyl)piperidine-1-carboxamide (C22); Maritinone (C38); 3-Methyl-benzofuran-2-carboxylic acid pyridin-4-ylamide (C21) and 5-(4-Ethyl-phenyl)-2-(1H-tetrazol-5-ylmethyl)-2H-tetrazole (C29) were organized using RS-Web Predictor tool. The metabolic sites on the compounds were indicated by circles on the chemical structure of the four ligands. The predictions of P450 Site of metabolism are shown in Table 3.

**Table 3:** The P450 Site of Metabolism (SOM) prediction results of the best four ligands.

Drug identifier	C22	C38	C21	C29
Names of P450 *iso*-enzymes	3-[3-(4-Fluorophenyl)-1,2,4-oxadiazol-5-yl]-N-(2-methylphenyl)piperidine-1-carboxamide	Maritinone	3-Methyl-benzofuran-2-carboxylic acid pyridin-4-ylamide	5-(4-Ethyl-phenyl)-2-(1H-tetrazol-5-ylmethyl)-2H-tetrazole
1A2				
2A6				
2B6				
2C8				
2C9				
2C19				
2D6				
2E1				
3A4				
Combined				

### 3.3 Drug-likeness and ADMET (absorption, distribution, metabolism, excretion, toxicity) analysis

The concept of drug-likeness has appeared as an approach that can screen the high-affinity ligands with acceptable ADMET (absorption, distribution, metabolism, excretion, toxicity) properties. In our study, small drug-like compounds: 3-[3-(4-Fluorophenyl)-1,2,4-oxadiazol-5-yl]-N-(2-methylphenyl)piperidine-1-carboxamide (C22); Maritinone (C38); 3-Methyl-benzofuran-2-carboxylic acid pyridin-4-ylamide (C21) and 5-(4-Ethyl-phenyl)-2-(1H-tetrazol-5-ylmethyl)-2H-tetrazole (C29) were subjected to Drug-likeness and ADMET analysis and all of them followed the Lipinski’s rule of five criteria: molecular weight (acceptable range: <500), number of hydrogen bond donors (acceptable range: ≤5), number of hydrogen bond acceptors (acceptable range: ≤10), lipophilicity (expressed as LogP, acceptable range: <5) and molar refractivity (40–130). C38 had the highest topological polar surface area (108.74 Å2) and C21 had the lowest polar surface area (55.13 Å2) although all of the five compounds satisfy the ideal value (20–130 Å2). The Ghose, Veber, Egan, Muegge rules are followed by all of the four compounds. The number of rotatable bonds, bioavailability scores, log S fell within the standard range for the four compounds (Table 4).

**Table 4:** The Drug-Likeness properties of the best four ligands.

Drug identifier	C22	C38	C21	C29
Drug Likeness Properties	3-[3-(4-Fluorophenyl)-1,2,4-oxadiazol-5-yl]-N-(2-methylphenyl)piperidine-1-carboxamide	Maritinone	3-Methyl-benzofuran-2-carboxylic acid pyridin-4-ylamide	5-(4-Ethyl-phenyl)-2-(1H-tetrazol-5-ylmethyl)-2H-tetrazole
Molecular weight	380.42 g/mol	374.34 g/mol	252.27 g/mol	256.27 g/mol
Concensus Log P_o_/w	3.77	2.88	2.55	1.39
Log S	−4.67	−4.77	−3.55	−2.90
Num. H-bond acceptors	5	6	3	6
Num. H-bond donors	1	2	1	1
Molar Refractivity	107.77	101.14	73.19	66.51
Lipinski	Yes; 0 violation	Yes; 0 violation	Yes; 0 violation	Yes; 0 violation
Ghose	Yes	Yes	Yes	Yes
Veber	Yes	Yes	Yes	Yes
Egan	Yes	Yes	Yes	Yes
Muegge	Yes	Yes	Yes	Yes
Bioavailability score	0.55	0.55	0.55	0.56
TPSA (Å^2^)	71.26 Å^2^	108.74 Å^2^	55.13 Å^2^	98.06 Å^2^
No of rotatable bonds	5	1	3	4

The relative ADMET profiles of screened ligands are described in Table 5. The evaluated pharmacokinetic data showed that all of the selected molecules had a high intestinal absorption rate and oral bioavailability. Each of the chosen molecules were capable of pervading Caco2 cell lines. C22 acted as P-glycoprotein substrate, P- glycoprotein inhibitor, and substrate for CYP3A4 cytochrome.

**Table 5:** ADMET Prediction of the best four ligands.

Drug identifier	C22	C38	C21	C29
Properties	3-[3-(4-Fluorophenyl)-1,2,4-oxadiazol-5-yl]-N-(2-methylphenyl)piperidine-1-carboxamide	Maritinone	3-Methyl-benzofuran-2-carboxylic acid pyridin-4-ylamide	5-(4-Ethyl-phenyl)-2-(1H-tetrazol-5-ylmethyl)-2H-tetrazole
Human Intestinal Absorption	Positive (0.99)	Positive (0.99)	Positive (0.97)	Positive (0.99)
Blood Brain Barrier	Positive (0.99)	Negative (0.71)	Positive (0.99)	Positive (0.98)
Caco-2	Positive (0.56)	Positive (0.69)	Positive (0.81)	Positive (0.71)
Human oral bioavailability	Positive (0.59)	Positive (0.63)	Positive (0.84)	Positive (0.60)
Subcellular localization	Mitochondria (0.70)	Mitochondria (0.90)	Mitochondria (0.44)	Mitochondria (0.81)
P-glycoprotein inhibitor	Positive (0.65)	Negative (0.83)	Negative (0.82)	Negative (0.94)
P-glycoprotein substrate	Positive (0.65)	Negative (0.96)	Negative (0.86)	Negative (0.56)
CYP3A4 substrate	Positive (0.65)	Negative (0.60)	Positive (0.51)	Negative (0.56)
CYP2C9 substrate	Positive (0.59)	Negative (0.80)	Negative (1.00)	Negative (0.80)
CYP2D6 substrate	Negative (0.83)	Negative (0.87)	Negative (0.90)	Negative (0.87)
CYP3A4 inhibition	Negative (0.52)	Negative (0.70)	Positive (0.79)	Negative (0.96)
CYP2C9 inhibition	Positive (0.52)	Positive (0.96)	Positive (0.74)	Negative (0.86)
CYP2C19 inhibition	Negative (0.52)	Positive (0.78)	Positive (0.86)	Negative (0.64)
CYP2D6 inhibition	Negative (0.91)	Negative (0.70)	Negative (0.57)	Negative (0.92)
CYP1A2 inhibition	Negative (0.72)	Positive (0.92)	Positive (0.97)	Positive (0.71)
Hepatotoxicity	Negative (0.58)	Positive (0.93)	Positive (0.95)	Negative (0.50)
Carcinogenicity (binary)	Negative (0.89)	Negative (0.65)	Negative (0.86)	Negative (0.91)

None of the ligands acted as CYP2D6 substrate or CYP2D6 inhibitor. Only C29 showed an inhibitory effect on CYP2C9 cytochrome. C22 acted as an inhibitor for CYP3A4, CYP2C19, CYP1A2 cytochrome but acted as a substrate for CYP2C9 cytochrome. C21 inhibited CYP3A4, CYP2C19, CYP1A2 cytochrome isoform but represented as a substrate for CYP3A4 isoform.

**Fig. 2:**
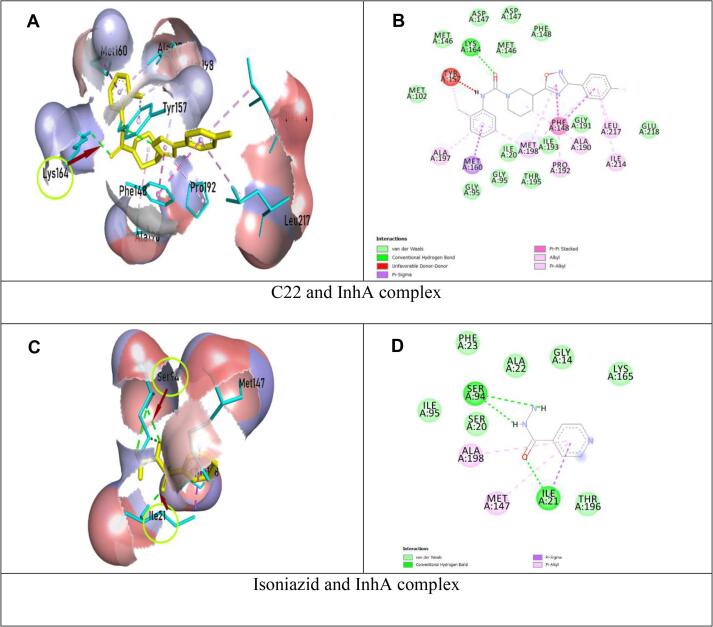
Schematic representation of InhA and drug (C22, Isoniazid) complex; On the left, ligands were in yellow color, parts of protein in cyan color, hydrogen bonds and interacting amino acids were shown with arrows and circle; On the right, two-dimensional image of InhA and drug (C22, Isoniazid) interaction were shown, green dotted line denoted hydrogen bonds. (For interpretation of the references to color in this figure legend, the reader is referred to the web version of this article.)

**Fig. 3:**
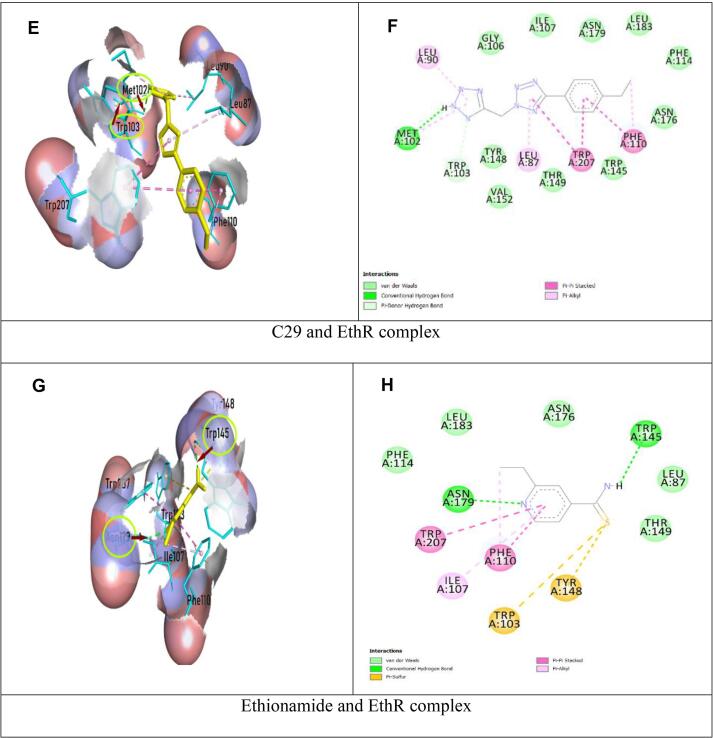
Schematic representation of EthR and drug (C29, Ethionamide) complex; On the left, ligands were in yellow color, parts of protein in cyan color, hydrogen bonds and interacting amino acids were shown with arrows and circle; On the right, two-dimensional image of EthR and drug (C29, Ethionamide) interaction were shown, green dotted line denoted hydrogen bonds. (For interpretation of the references to color in this figure legend, the reader is referred to the web version of this article.)

All selected ligands were non-carcinogenic. C22 and C29 were non-hepatotoxic. The best ligands and control drugs with respective receptors (C22 and InhA complex, Isoniazid and InhA complex, C29 and EthR complex, Ethionamide and EthR complex) were depicted in Fig. 2, Fig. 3.

### 3.4 Molecular Dynamics Simulation:

**Fig. 4:**
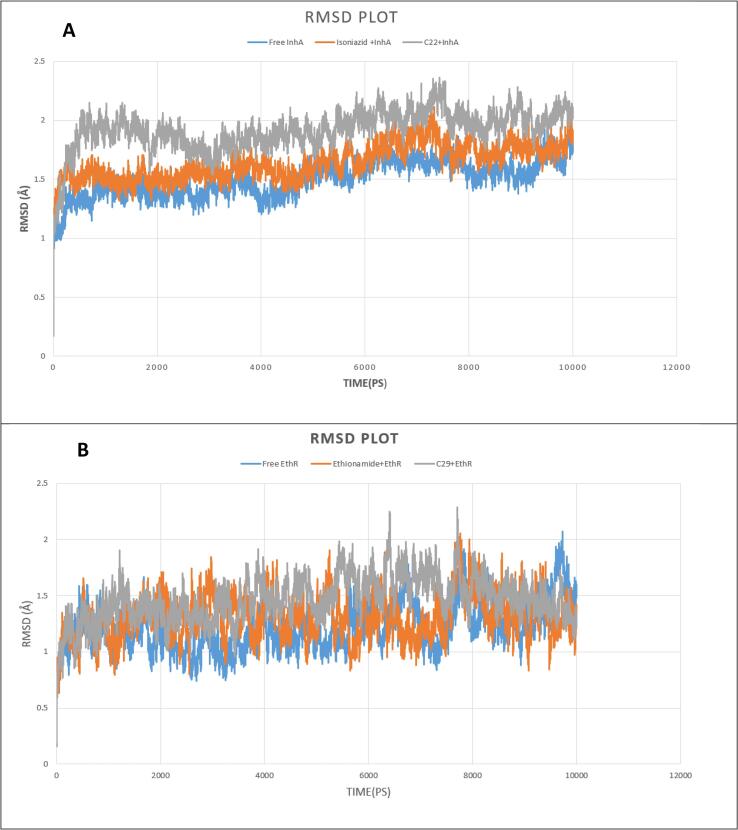
10 ns Molecular Dynamics Simulation (MDS) RMSD of Free protein and bounded protein; (A, B) Free protein (InhA and EthR) in blue color, Control drugs (Isoniazid and Ethionamide) and protein in orange color, Selected drug (C22 + C29) and protein in grey color. (For interpretation of the references to color in this figure legend, the reader is referred to the web version of this article.)

In our study, Molecular Dynamics Study was performed to assess the conformational stability of protein–ligand complexes as well as InhA and EthR receptors. The overall conformational similarity between drugs (control, C22, and C29) with target proteins was compared assessing Root Mean Square deviation (RMSD). As represented in Fig. 4(A), RMSD of Free InhA protein c3ontinued stability between 1 ns and 5 ns timescale at about 1.4 Å and 5 ns (nanosecond) to 9 ns at about 1.7 Å. (Isoniazid + InhA) complex displayed steadiness in RMSD between 1 ns and 5 ns timescale at around 1.5 Å and 8 ns to till the end of run at around 1.8 Å. (C22 + InhA) complex held stable backbone stability from 3 ns to 7 ns, with RMSD around 2 Å. Free EthR protein, (Ethionamide + EthR) protein–ligand complex and (C29 + EthR) complex showed a backbone RMSD under 2 Å, which suggested minor structural changes. Free EthR protein remained stable between 1 ns and 6 ns, exhibiting RMSD around 1.2 Å. (Ethionamide + EthR) complex also showed stability from 1 ns to 6 ns timeline, with an RMSD backbone of 1.2 Å, then exhibiting small increased RMSD. On the other hand, RMSD of (C29 + EthR) complex persisted stable from 1 ns to 5 ns timeline at about 1.5 Å, then after slight fluctuation, remained stable from 8 ns to the end of run-on average 1.4 Å.

**Fig. 5:**
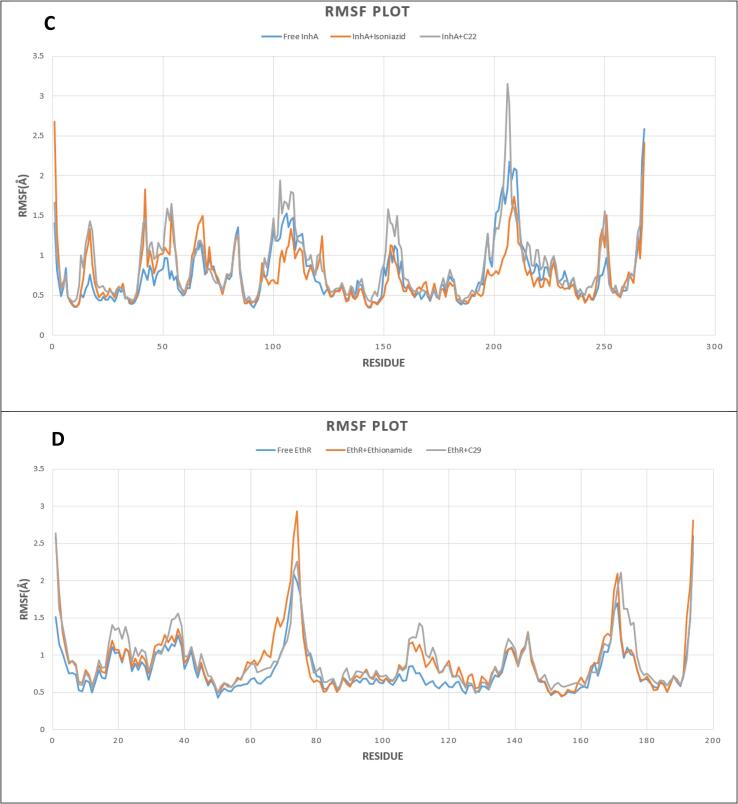
RMSF outline of Free protein and bounded protein; (C, D) Free protein (InhA and EthR) in blue color, Control drugs (Isoniazid and Ethionamide) and protein in orange color, Selected drug (C22 + C29), and protein in grey color. (For interpretation of the references to color in this figure legend, the reader is referred to the web version of this article.)

Root Mean Square Fluctuation (RMSF) analysis per residue for backbone atoms was conducted to assess changes in the conformation of Cα backbone of the systems. From Fig. 5(C), Free InhA, (Isoniazid + InhA), (C22 + InhA) complexes mostly had RMSF from 0.4 to 2 Å that indicated close conformational contact between protein and ligands. Nevertheless, the higher fluctuation of RMSF between 205 and 211 residues confirmed the presence of loop within this region. Our MDS study showed that the RMSF of Free EthR, (EthR + Ethionamide), (EthR + C29) complexes fluctuated mostly between 0.5 and 1.5 Å, indicating close contact between the active pocket of receptors and drugs. However, higher fluctuation from 73 to 75 and 170 to 172 amino acid residues indicated that the free protein and its complexes were within the loop regions.

**Fig. 6:**
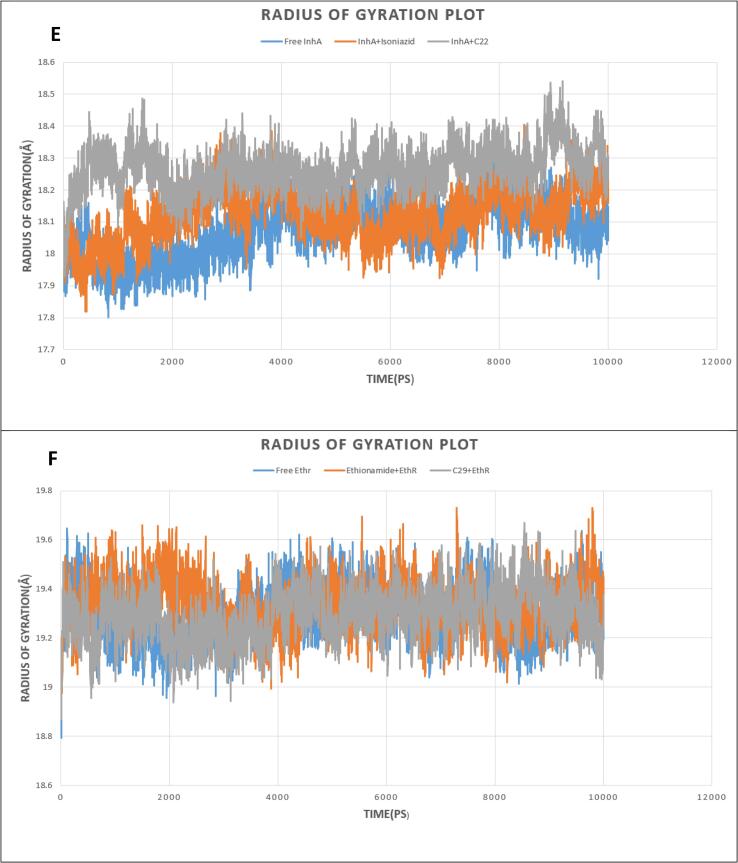
Analysis of Radius of Gyration (Rg); (E, F) Free protein (InhA and EthR) in blue color, Control drugs (Isoniazid and Ethionamide) and protein in orange color, Selected drug (C22 + C29) and protein in grey color. (For interpretation of the references to color in this figure legend, the reader is referred to the web version of this article.)

Radius of Gyration with time was calculated to assess the change of compactness after ligand binding with receptors. From Fig. 6(E), the Rg of Free InhA was reported between 17.9 and 18.2 Å; (InhA + Isoniazid) complex showed Rg between 17.9 and 18.3 Å; (InhA + C22) complex continue Rg between 18.1 and 18.4 Å through the simulation. This evaluation proved ligand binding did not affect the compactness of the protein. On the other hand, from Fig. 6(F), it was evident Free EthR had stable Rg value around 19.3 Å from 2 to 8 ns; (EthR + Ethionamide) complex remained stable Rg about 19.4 Å between 5 ns to till the end of simulation and (EthR + C29) complex kept stability from 5 to 9 ns, with Rg around 19.3 Å. This confirmed ligand binding with the respective receptor did not cause structural instability to proteins.

## 4 Discussion

In our study, virtual screening of high-affinity ligands was accomplished by two stages of molecular docking analysis. As for the initial docking evaluation, fifty ligands were docked separately against the InhA and EthR receptor proteins through the DockThor server [34]. Both InhA and EthR proteins are associated in vital function in which InhA is involved in the synthesis of mycolic acids, essential part of mycobacterial cell wall and EthR, a repressor of monooxygenase EthA, brings about resistance to Ethionamide [50], [51]. For both InhA and EthR proteins, both drug-like compounds had a lower affinity score than their respective control drugs. Subsequently, Autodock Vina software was used for calculating binding energy and bound conformation in which two ligands for InhA protein and two ligands for EthR protein were selected based on their lower binding score [35].

Cytochrome P450 (CYPs) enzymes are metabolic enzymes, responsible for the biotransformation of ~90% FDA certified drugs [52]. The oxidative metabolism of drugs in phase I is achieved by the CYP system [53], [54]. Nine of the isozymes under the CYP system scanned for the prediction of the metabolically vulnerable points using RS-WebPredictor tool [37]. This tool helps to predict the regioselectivity of isozyme-specific CYP mediated xenobiotic metabolism on any set of user-submitted molecules [55]. C22, C38, C21, C29 drugs displayed several sites of metabolism for CYP1A2, 2A6, 2B6, 2C8, 2C19, 2E1, 3A4, 2C9, 2D6 isoforms that suggested a satisfactory result.

Drug-likeness is an approach that qualitatively evaluates the solubility, chemical stability, bioavailability and distribution profile of a drug like molecule [56], [57]. Lipinski’s Rule of 5, a rule of thumb was established to evaluate the ‘drugability’ of new chemical entities having certain pharmacological or biological activity [58]. Our selected four compounds: 3-[3-(4-Fluorophenyl)-1,2,4-oxadiazol-5-yl]-N-(2-methylphenyl)piperidine-1-carboxamide (C22); Maritinone (C38); 3-Methyl-benzofuran-2-carboxylic acid pyridin-4-ylamide (C21) and 5-(4-Ethyl-phenyl)-2-(1H-tetrazol-5-ylmethyl)-2H-tetrazole (C29) followed the Lipinski’s rule of five criteria without violating any of its parameters. In addition to following the Lipiniski rule, they followed the Ghose, Veber, Egan, and Muegge rules as well. Given the standard values, our four compounds pass solubility (Log S), bioavailability score, TPSA (Å2), rotatable bond number.

Human Intestinal Absorption (HIA) is a pharmacokinetic process that determines the effectivity of intestinal absorption or bioavailability of a drug upon oral administration, an anticipated route of drug administration [59]. Our four ligands showed a high rate of intestinal absorption and oral bioavailability. Caco-2 cell line, a prominent substitute to human intestinal epithelium (mucosa) is one of the in-vitro models to determine in vivo human intestinal absorption of drug molecules due to their morphological and functional resemblances with human enterocytes [60]. All of our potential drug candidates showed the ability to penetrate Caco-2 cell line. C22 acted as P-gp substrates as well as inhibitors. On the other hand, C38, C21, C29 were neither P-gp substrates nor P-gp inhibitors.

Since the occurrence of hepatotoxicity caused by anti-TB is one of the frequent reasons behind the termination of anti-TB products, it is important to evaluate the possible hepatotoxicity of novel drugs [61]. As well as a carcinogenicity test is necessary to classify a tumorigenic potentiality of drugs to assess the relevant risk in humans [62]. Only two ligands, C22 and C29 passed two of the criteria: hepatotoxicity, and carcinogenicity as they showed negative results.

To validate the conformational stability of our proposed drug upon binding with receptors, Molecular Dynamics Simulation was performed. Root Mean Square deviation (RMSD) analysis showed that binding of both control drugs and proposed drug C29 did not induce structural instability to the proteins because the reported RMSD change after ligand binding remained under 2 Å for both receptors. A thorough study of Root Mean Square Fluctuation (RMSF) curve revealed our tested compounds kept close contact with their active sites which was evident from their small range fluctuation under 1.5 Å and 2 Å for EthR and InhA complexes respectively. Though, because of loop regions on receptor proteins, greater fluctuation of RMSF was seen [63]. Radius of Gyration analysis showed that our proposed drug C29 and C22 caused less fluctuation when bound to their respective receptors relative to control drugs. This confirmed C29 and C22 did not cause instability to receptors. We compared our proposed drugs C29 and C22 with control drugs (Ethionamide and Isoniazid) through Molecular Dynamics Simulation (MDS), in which C29, C22 protein complexes represented stability throughout 10 ns simulation.

Starting from the preliminary docking analysis to Molecular Dynamics Simulation, our candidate compounds were thoroughly examined. At the stage of docking, our selected four candidates showed greater binding affinity in comparison to the control drugs. Additionally, the best four compounds examined for analyzing pharmacokinetics and pharmacodynamics properties and they showed promising results in drug-likeness and ADMET profiling. In all aspects, our chosen 3-[3-(4-Fluorophenyl)-1,2,4-oxadiazol-5-yl]-N-(2-methylphenyl) piperidine-1-carboxamide and 5-(4-Ethyl-phenyl)-2-(1H-tetrazol-5-ylmethyl)-2H-tetrazole showed satisfactory result than other two of the ligands. To conclude, 3-[3-(4-Fluorophenyl)-1,2,4-oxadiazol-5-yl]-N-(2-methylphenyl) piperidine-1-carboxamide and 5-(4-Ethyl-phenyl)-2-(1H-tetrazol-5-ylmethyl)-2H-tetrazole could be chosen as the most effective and promising candidates for as anti-TB drug.

Despite the fact that drug repurposing earned tremendous success, there are some caveats to the in-silico methodology. Like, one of the downsides of molecular docking is the provision of appropriate scoring functions and algorithms, which may otherwise jeopardize molecular screening [64]. Although our entire approach was based on computational tools, the findings were validated by dynamic molecular simulation and pharmacokinetic profiling of drug-like compounds.

Our study aims to identify the linkage between tuberculosis and already established antibacterial drugs applying several bioinformatics tools. This methodology can evade the experimental hurdles of screening thousands of ligands for tuberculosis. Integration of in silico and experimental approaches led to discovery of Lidocaine, Methotrexate, Mifepristone and Zidovudine as a potent therapeutic against Arrhythmia, Arrhythmia, Cushing’s syndrome and HIV (human immunodeficiency virus) respectively [65], [66]. To prove the authenticity of our findings, in vivo research involving animal model is required. The curated dataset can be a great deal of interest to those researchers working in this field to find novel anti-TB drugs.

## 5 Conclusion

The number of people dying annually of TB is growing rapidly due to multiple drug resistance scenarios around the world. This situation demands newer anti-TB drugs to deal with the crisis. Drug repurposing is an easier and cheaper option to look for novel candidates as anti-TB drugs using different computational tools. The main objective of this study is to a find novel inhibitor against fast-mutating anti-TB drug targets. Our work incorporates pharmacophore analysis, ADMET profiling, two-step molecular docking, followed by 10 ns Molecular Dynamics Simulation. Drugs with greater binding affinity than the control drugs are considered for determining Drug-likeness and ADMET analysis to evaluate their bioavailability and toxicity. Two of our screened compounds: 3-[3-(4-Fluorophenyl)-1,2,4-oxadiazol-5-yl]-N-(2-methylphenyl) piperidine-1-carboxamide and 5-(4-Ethyl-phenyl)-2-(1H-tetrazol-5-ylmethyl)-2H-tetrazole showed promising results with higher binding affinity with respective receptors and standard pharmacophoric properties. Molecular Dynamics Simulation study including RMSD, RMSF, Rg analysis confirmed their binding stability with respective proteins throughout the simulation timeline. Our present work could be productive in discovering potential therapeutics against multiple drug resistant tuberculosis, stating that substantial in vitro and in vivo experiments are needed to prove our hypothetical study.

## Declaration of Competing Interest

The authors declare that they have no known competing financial interests or personal relationships that could have appeared to influence the work reported in this paper.

## References

[1] Smith I. (2003). Mycobacterium tuberculosis pathogenesis and molecular determinants of virulence. Clin Microbiol Rev.

[2] Gordon S.V., Parish T. (2018). Microbe profile: mycobacterium tuberculosis: humanity's deadly microbial foe. Microbiology.

[3] Orcau À., Caylà J.A., Martínez J.A. (2011). Present epidemiology of tuberculosis. Prevention and control programs. Enferm Infecc Microbiol Clin.

[4] Cole S.T. (1999). Learning from the genome sequence of Mycobacterium tuberculosis H37Rv. FEBS Lett.

[5] Churchyard G., Kim P., Shah N.S., Rustomjee R., Gandhi N., Mathema B., Dowdy D., Kasmar A., Cardenas V. (2017). What we know about tuberculosis transmission: an overview. J. Infect. Di..

[6] Bussi C., Gutierrez M.G. (2019). Mycobacterium tuberculosis infection of host cells in space and time. FEMS Microbiol Rev.

[7] Eruslanov E.B., Majorov K.B., Orlova M.O., Mischenko V.V., Kondratieva T.K., Apt A.S. (2004). Lung cell responses to M. tuberculosis in genetically susceptible and resistant mice following intratracheal challenge. Clin Exp Immunol.

[8] Ahmad, S., 2011. Pathogenesis, immunology, and diagnosis of latent Mycobacterium tuberculosis infection. Clin Develop Immunol, 2011. 10.1155/2011/814943 PMC3017943 21234341

[9] Zaman K. (2010). Tuberculosis: a global health problem. J Health Popul Nutr.

[10] Madhavaram M., Nampally V., Gangadhari S., Palnati M.K., Tigulla P. (2019). High-throughput virtual screening, ADME analysis, and estimation of MM/GBSA binding-free energies of azoles as potential inhibitors of Mycobacterium tuberculosis H37Rv. J Recept Signal Transd.

[11] Loddenkemper, R., Lipman, M. and Zumla, A., 2016. Clinical aspects of adult tuberculosis. Cold Spring Harbor perspectives in medicine, 6(1), p.a017848.Muñoz, L., Stagg, H. and Abubakar, I. (2015) “Diagnosis and Management of Latent Tuberculosis Infection: Table 1.”, Cold Spring Harbor Perspectives in Medicine, 5(11), p. a017830. doi: 10.1101/cshperspect.a017830. 10.1101/cshperspect.a017848 PMC4691808 25659379

[12] Muñoz L., Stagg H.R., Abubakar I. (2015). Diagnosis and management of latent tuberculosis infection: Table 1. Cold Spring Harbor Perspect Med.

[13] Daffe M, Laneelle MA, Asselineau C, Levy-Frebault V, David H. 1983. Taxonomic value of mycobacterial fatty acids: proposal for a method of analysis. In Annales de microbiologie (Vol. 134, No. 2, p. 241). 6651121

[14] Hoffmann C., Leis A., Niederweis M., Plitzko J.M., Engelhardt H. (2008). Disclosure of the mycobacterial outer membrane: cryo-electron tomography and vitreous sections reveal the lipid bilayer structure. Proc Natl Acad Sci.

[15] Alderwick LJ, Birch HL, Mishra AK, Eggeling L, Besra GS. Structure, function and biosynthesis of the Mycobacterium tuberculosis cell wall: arabinogalactan and lipoarabinomannan assembly with a view to discovering new drug targets; 2007. 10.1042/BST0351325 17956343

[16] Besra G.S., Kremer L. (2002). Current status and future development of antitubercular chemotherapy. Expert Opin Invest Drugs.

[17] Jarlier V., Nikaido H. (1994). Mycobacterial cell wall: structure and role in natural resistance to antibiotics. FEMS Microbiol Lett.

[18] Brennan P.J., Nikaido H. (1995). The envelope of mycobacteria. Annu Rev Biochem.

[19] Dubnau, E., Chan, J., Raynaud, C., Mohan, V.P., Lanéelle, M.A., Yu, K., Quémard, A., Smith, I, Daffé, M., 2000. Oxygenated mycolic acids are necessary for virulence of Mycobacterium tuberculosis in mice. Mol Microbiol, 36(3), pp.630–7. 10.1046/j.1365-2958.2000.01882.x 10844652

[20] Dkhar H.K., Nanduri R., Mahajan S., Dave S., Saini A., Somavarapu A.K. (2014). Mycobacterium tuberculosis keto-mycolic acid and macrophage nuclear receptor TR4 modulate foamy biogenesis in granulomas: a case of a heterologous and noncanonical ligand-receptor pair. J Immunol.

[21] Dessen A., Quemard A., Blanchard J., Jacobs W., Sacchettini J. (1995). Crystal structure and function of the isoniazid target of Mycobacterium tuberculosis. Science.

[22] Engohang-Ndong J. et al. (2003) EthR, a repressor of the TetR/CamR family implicated in ethionamide resistance in mycobacteria, octamerizes cooperatively on its operator, Mol Microbiol, 51(1), pp. 175–88. doi: 10.1046/j.1365-2958.2003.03809.x. 10.1046/j.1365-2958.2003.03809.x 14651620

[23] Hameed H.M.A., Islam M.M., Chhotaray C., Wang C., Liu Y., Tan Y. (2018). Molecular targets related drug resistance mechanisms in MDR-, XDR-, and TDR-Mycobacterium tuberculosis strains. Front Cell Infect Microbiol.

[24] Willand N., Dirié B., Carette X., Bifani P., Singhal A., Desroses M. (2009). Synthetic EthR inhibitors boost antituberculous activity of ethionamide. Nat Med.

[25] Takayama K., Wang L., David H. (1972). Effect of Isoniazid on the In Vivo Mycolic Acid Synthesis, Cell Growth, and Viability of Mycobacterium tuberculosis. Antimicrob Agents Chemother.

[26] Volmink, J. and Garner, P., 2007. Directly observed therapy for treating tuberculosis. Cochrane Database of System Rev, (4). 10.1002/14651858.CD003343 11687192

[27] Dookie N., Rambaran S., Padayatchi N., Mahomed S., Naidoo K. (2018). Evolution of drug resistance in Mycobacterium tuberculosis: a review on the molecular determinants of resistance and implications for personalized care. J Antimicrob Chemother.

[28] Gygli S.M., Borrell S., Trauner A., Gagneux S. (2017). Antimicrobial resistance in Mycobacterium tuberculosis: mechanistic and evolutionary perspectives. FEMS Microbiol Rev.

[29] Lienhardt, C., Raviglione, M., Spigelman, M., Hafner, R., Jaramillo, E., Hoelscher, M., Zumla, A. and Gheuens, J., 2012. New drugs for the treatment of tuberculosis: needs, challenges, promise, and prospects for the future. J Inf Dis, 205(suppl_2), pp.S241-S249. 10.1093/infdis/jis034 22448022

[30] Velayati A.A., Farnia P., Farahbod A.M. (2016). Overview of drug-resistant tuberculosis worldwide. Int J Mycobacteriol.

[31] World Health Organization (2017). END TB global tuberculosis report 2017.

[32] Schrödinger LLC. The PyMOL Molecular Graphics System, Version 2.0 Schrödinger, LLC (2017). Google Scholar There is no corresponding record for this reference.

[33] Guex N., Peitsch M.C. (1997). SWISS-MODEL and the Swiss-Pdb Viewer: an environment for comparative protein modeling. Electrophoresis.

[34] Guedes I, Krempser E, Dardenne E. (2017) DockThor 2 . 0 : a Free Web Server for Protein-Ligand Virtual Screening, Semanticscholar.org. Available at: https://www.semanticscholar.org/paper/DockThor-2-.-0-:-a-Free-Web-Server-for-Virtual-Guedes-Krempser/4b9eb90fdcc7193358c6b214def17eb490896b10?p2df (Accessed: 22 November 2020).

[35] Morris G.M., Huey R., Lindstrom W., Sanner M.F., Belew R.K., Goodsell D.S. (2009). AutoDock4 and AutoDockTools4: Automated docking with selective receptor flexibility. J Comput Chem.

[36] Tian, W. et al. (2018) CASTp 3.0: computed atlas of surface topography of proteins, Nucl Acids Res, 46(W1), pp. W363-W367. doi: 10.1093/nar/gky473. 10.1093/nar/gky473 PMC6031066 29860391

[37] Zaretzki J., Bergeron C., Huang T.-w., Rydberg P., Swamidass S.J., Breneman C.M. (2013). RS-WebPredictor: a server for predicting CYP-mediated sites of metabolism on drug-like molecules. Bioinformatics.

[38] Lipinski C.A., Lombardo F., Dominy B.W., Feeney P.J. (1997). Experimental and computational approaches to estimate solubility and permeability in drug discovery and development settings. Adv Drug Deliv Rev.

[39] Ghose A., Viswanadhan V., Wendoloski J. (1999). A knowledge-based approach in designing combinatorial or medicinal chemistry libraries for drug discovery. 1. A qualitative and quantitative characterization of known drug databases. J Comb Chem.

[40] Veber D.F., Johnson S.R., Cheng H.-Y., Smith B.R., Ward K.W., Kopple K.D. (2002). Molecular properties that influence the oral bioavailability of drug candidates. J Med Chem.

[41] Muegge I., Heald S., Brittelli D. (2001). Simple selection criteria for drug-like chemical matter. J Med Chem.

[42] Daina A., Michielin O., Zoete V. (2017). SwissADME: a free web tool to evaluate pharmacokinetics, drug-likeness and medicinal chemistry friendliness of small molecules. Sci Rep.

[43] Yang, H., Lou, C., Sun, L., Li, J., Cai, Y., Wang, Z., Li, W., Liu, G. and Tang, Y., 2019. admetSAR 2.0: web-service for prediction and optimization of chemical ADMET properties. Bioinformatics, 35(6), pp. 1067–69. 10.1093/bioinformatics/bty707 30165565

[44] Shawan M.M.A.K., Halder S.K., Hasan M.A. (2021). Luteolin and abyssinone II as potential inhibitors of SARS-CoV-2: an in silico molecular modeling approach in battling the COVID-19 outbreak. Bull Natl Res Centre.

[45] Xu L., Jiang W., Jia H., Zheng L., Xing J., Liu A. (2020). Discovery of multitarget-directed ligands against influenza a virus from compound yizhihao through a predictive system for compound-protein interactions. Front Cell Infect Microbiol.

[46] Phillips J.C., Braun R., Wang W., Gumbart J., Tajkhorshid E., Villa E. (2005). Scalable molecular dynamics with NAMD. J Comput Chem.

[47] MacKerell A.D., Bashford D., Bellott M., Dunbrack R.L., Evanseck J.D., Field M.J. (1998). Allatom empirical potential for molecular modeling and dynamics studies of proteins. J Phys Chem B.

[48] Humphrey W., Dalke A., Schulten K. (1996). VMD: visual molecular dynamics. J Mol Graph.

[49] Jo S., Kim T., Iyer V.G., Im W. (2008). CHARMM-GUI: a web-based graphical user interface for CHARMM. J Comput Chem.

[50] Duan X., Xiang X., Xie J. (2014). Crucial components of mycobacterium type II fatty acid biosynthesis (Fas-II) and their inhibitors. FEMS Microbiol Lett.

[51] DeBarber A.E., Mdluli K., Bosman M., Bekker L.-G., Barry C.E. (2000). Ethionamide activation and sensitivity in multidrug-resistant Mycobacterium tuberculosis. Proc Natl Acad Sci.

[52] Nebert D., Russell D. (2002). Clinical importance of the cytochromes P450. Lancet.

[53] Phang-Lyn, S. and Llerena, V. (2020) “Biochemistry, Biotransformation”, StatPearls Publishing, p. Available at: https://www.ncbi.nlm.nih.gov/books/NBK544353/ (Accessed: 22 November 2020). 31335073

[54] Zanger U.M., Turpeinen M., Klein K., Schwab M. (2008). Functional pharmacogenetics/genomics of human cytochromes P450 involved in drug biotransformation. Anal Bioanal Chem.

[55] Zanger U., Schwab M. (2013). Cytochrome P450 enzymes in drug metabolism: regulation of gene expression, enzyme activities, and impact of genetic variation. Pharmacol Ther.

[56] Sarkar B., Islam S.S., Ullah M.A., Hossain S., Prottoy M.N.I., Araf Y. (2019). Computational assessment and pharmacological property breakdown of eight patented and candidate drugs against four intended targets in Alzheimer’s disease. Adv Biosci Biotechnol.

[57] Ullah M.A., Johora F.T., Sarkar B., Araf Y., Rahman MD.H. (2020). Curcumin analogs as the inhibitors of TLR4 pathway in inflammation and their drug like potentialities: a computer-based study. J Recept Signal Transduction.

[58] Lipinski C. (2000). Drug-like properties and the causes of poor solubility and poor permeability. J Pharmacol Toxicol Methods.

[59] Radchenko E.V., Dyabina A.S., Palyulin V.A., Zefirov N.S. (2016). Prediction of human intestinal absorption of drug compounds. Russ Chem Bull.

[60] Wang N.-N., Dong J., Deng Y.-H., Zhu M.-F., Wen M., Yao Z.-J. (2016). ADME properties evaluation in drug discovery: prediction of caco-2 cell permeability using a combination of NSGA-II and boosting. J Chem Inf Model.

[61] Ramappa V., Aithal G. (2013). Hepatotoxicity related to anti-tuberculosis drugs: mechanisms and management. J Clin Exp Hepatol.

[62] Todd Bourcier, D. (2015) “Improving Prediction of Carcinogenicity to Reduce, Refine, and Replace the Use of Experimental Animals”, Journal of the American Association for Laboratory Animal Science: JAALAS, 54(2), p. 163. Available at: https://www.ncbi.nlm.nih.gov/pmc/articles/PMC4382620/ (Accessed: 22 November 2020). PMC4382620 25836962

[63] Brigo A., Lee K.W., Iurcu Mustata G., Briggs J.M. (2005). Comparison of multiple molecular dynamics trajectories calculated for the drug-resistant HIV-1 Integrase T66I/M154I catalytic domain. Biophys J.

[64] Sacan A., Ekins S., Kortagere S. (2012). Applications and limitations of in silico models in drug discovery. Bioinform Drug Disc.

[65] March-Vila E., Pinzi L., Sturm N., Tinivella A., Engkvist O., Chen H. (2017). On the integration of in silico drug design methods for drug repurposing. Front Pharmacol.

[66] Akhoon BA, Tiwari H, Nargotra A. 2019. In silico drug design methods for drug repurposing. In In Silico Drug Design (pp. 47-84). Academic Press.

